# Effective Rheology of Two-Phase Flow in Three-Dimensional Porous Media: Experiment and Simulation

**DOI:** 10.1007/s11242-017-0874-4

**Published:** 2017-06-13

**Authors:** Santanu Sinha, Andrew T. Bender, Matthew Danczyk, Kayla Keepseagle, Cody A. Prather, Joshua M. Bray, Linn W. Thrane, Joseph D. Seymour, Sarah L. Codd, Alex Hansen

**Affiliations:** 1 Beijing Computational Science Research Center, 10 East Xibeiwang Road, Haidian District, Beijing, 100193 China; 2 PoreLab, Department of Physics, Norwegian University of Science and Technology, NTNU, 7491 Trondheim, Norway; 3 Department of Mechanical and Industrial Engineering, Montana State University, Bozeman, MT USA; 4 Department of Chemical and Biological Engineering, Montana State University, Bozeman, MT USA

**Keywords:** Dynamical pore network model, Reconstructed porous media, Two-phase flow experiment, Steady-state two-phase flow

## Abstract

We present an experimental and numerical study of immiscible two-phase flow of Newtonian fluids in three-dimensional (3D) porous media to find the relationship between the volumetric flow rate (*Q*) and the total pressure difference ($\Delta P$) in the steady state. We show that in the regime where capillary forces compete with the viscous forces, the distribution of capillary barriers at the interfaces effectively creates a yield threshold ($P_t$), making the fluids reminiscent of a Bingham viscoplastic fluid in the porous medium. In this regime, *Q* depends quadratically on an excess pressure drop ($\Delta P-P_t$). While increasing the flow rate, there is a transition, beyond which the overall flow is Newtonian and the relationship is linear. In our experiments, we build a model porous medium using a column of glass beads transporting two fluids, deionized water and air. For the numerical study, reconstructed 3D pore networks from real core samples are considered and the transport of wetting and non-wetting fluids through the network is modeled by tracking the fluid interfaces with time. We find agreement between our numerical and experimental results. Our results match with the mean-field results reported earlier.

**Electronic supplementary material:**

The online version of this article (doi:10.1007/s11242-017-0874-4) contains supplementary material, which is available to authorized users.

## Introduction

The simultaneous flow of two immiscible fluids in porous media, otherwise known as two-phase flow (Bear 1972; Dullien 1992), is getting increasing attention of both the scientific and industrial communities. These flows are encountered in many industrial and geophysical applications, such as carbon sequestration and oil recovery, groundwater management, blood flow in capillary vessels, catalyst supports used in automotive industry, bubble generation in microfluidics and many more. Extensive study of hydrodynamics in porous media is crucial for the development of these applied processes. While single-phase flows have been well characterized, many phenomena of steady-state immiscible two-phase flows in porous media are still not adequately understood.

In single-phase flow of Newtonian fluids, the macro-scale pressure gradient ($\Delta P$) over a porous medium scales linearly with the superficial fluid velocity governed by the Darcy’s law (Darcy 1856; Whitaker 1986). This is also true in the case of two-phase flows at high fluid velocities when capillary forces are negligible. Here, the flow in the pores generally has low Reynolds number and inertial effects are neglected. However, at slow velocities when the capillary forces are comparable with the viscous forces, the total pressure gradient in the steady state does not scale linearly with the flow rate. In a fundamental experiment of two-phase flow of air and water–glycerol mixture in a two-dimensional (2D) model porous media made of glass beads in Hele-Shaw cell (Tallakstad et al. 2009a, b), it was observed that $\Delta P$ scales with the flow rate by a power law with an exponent 0.54. In a different experiment of two-phase flow in a three-dimensional (3D) porous medium, similar power law scaling between *Q* and $\Delta P$ was observed, but the exponents were found to vary in the range between 0.45 and 0.3 depending on the saturation (Rassi et al. 2011). To investigate this scaling between $\Delta P$ and *Q*, Sinha and Hansen (2012) and Sinha et al. (2013) performed mean-field calculations and numerical simulations based on a pore-network model and derived a generalized Darcy equation for the steady-state two-phase flow of immiscible Newtonian fluids. They have shown that the capillary barriers at the interfaces between the fluids effectively create an effective yield threshold, making the fluids reminiscent of a Bingham viscoplastic fluid in the porous medium with an overall threshold pressure $P_t$ in the system below which there is no flow. It was shown that there are at-least two flow regimes, for small *Q* when blocked pores keep opening up with the increase in the pressure drop, *Q* depends quadratically on ($\Delta P-P_t$). For large *Q*, when all the pores have overcome the capillary barriers, *Q* become linear with $\Delta P$. For a single capillary tube with a narrow pore throat, the average flow rate $\langle q\rangle$ through one tube above the capillary barrier ($p_t$) can be obtained for the steady-state conditions by integrating the instantaneous linear two-phase flow equation over the whole tube. The problem becomes equivalent to a forced over-damped oscillator, providing a threshold pressure followed by a square-root singularity given by Sinha et al. (2013),

1 $$\begin{aligned} \displaystyle \left<q\right> = - \sigma _0\ \mathrm{sgn}(\Delta p) \left\{ \begin{array}{l@{\quad }l} \sqrt{\Delta p^2-p_t^2} &{} \hbox {if } |\Delta p| > p_t,\\ 0 &{} \hbox {if }|\Delta p| \le p_t,\\ \end{array}\right. \end{aligned}$$

where $\mathrm{sgn}()$ is the sign function and $\sigma _0$ contains the terms related to permeability. Close to the threshold, $(|\Delta p|-p_t) \ll p_t$, therefore $\sqrt{\Delta p^2 -p_t^2} \approx \sqrt{2p_t} \sqrt{|\Delta p|-p_t}$ and the average flow $\langle q\rangle \sim \sqrt{|\Delta p|-p_t}$. The square-root relationship here is a consequence of any quadratic extremum in the pore radius, which leads to a saddle-node bifurcation (Strogatz 1994), and therefore does not depend on any specific sinusoidal shape of the pore. $\sigma _0$ contains the terms related to the permeability of the single tube which depends on the cross section. For Poiseuille flow in a circular cross section, $\sigma _0=(\pi r_0^4)/(8l\mu _p)$ where $r_0$ and *l* are the radius and length of the tube, and $\mu _p$ is the effective viscosity of the fluids inside the tube (Langglois 1964; Jia et al. 2008). This relationship leads to the nonlinear conductivity of a single capillary given by $\sigma (\Delta p)=-\mathrm{d}\langle q\rangle /\mathrm{d}(\Delta p)$ in the steady state.^1^ Using this expression of $\sigma (\Delta p)$ for each link, a mean-field theory was developed to find the relationship of the total flow rate for a homogeneous disordered network (Sinha and Hansen 2012). For a spatially uncorrelated distribution of threshold pressures, one obtains

2 $$\begin{aligned} Q = -C\frac{A}{L}\frac{K(S_\mathrm{nw})}{\mu (S_\mathrm{nw})}\mathrm{sgn}\left( \Delta P\right) \left\{ \begin{array}{l@{\quad }l} \left( |\Delta P|-P_t(S_\mathrm{nw})\right) ^2 &{} \hbox {if }|\Delta P| > P_t,\\ 0 &{} \hbox {if }|\Delta P| \le P_t,\\ \end{array}\right. \end{aligned}$$

where *L* is length of the network, *C* is a constant with units of inverse pressure and $K(S_\mathrm{nw})$ is the effective permeability as a function of the non-wetting fluid saturation $S_\mathrm{nw}$. $\mu$ is the effective viscosity of the system given by $\mu =\mu _\mathrm{nw}S_\mathrm{nw}+\mu _\mathrm{w}(1-S_\mathrm{nw})$ where $\mu _\mathrm{w}$ and $\mu _\mathrm{nw}$ are the viscosities of the wetting and the non-wetting fluids, respectively. For the high flow rates, there is a transition, beyond which the two-phase flow rate varies linearly with the total pressure drop. These two flow regimes were also verified with extensive numerical simulations (Sinha and Hansen 2012). However, the simulations were performed only for regular 2D networks of disordered tubes and not for 3D pore networks.

The appearance of a threshold pressure ($P_t$) and the quadratic dependence of *Q* on $(\Delta P - P_t)$ in the steady-state two-phase flow of Newtonian fluids are reminiscent to the single-phase flow of Bingham viscoplastic fluid in a porous medium. A Bingham viscoplastic fluid is a yield stress fluid which flow like liquid when the applied stress across the fluid is higher than a critical value ($\tau _c$), the yield stress, below which it behaves as a solid. The rheology of a simple yield stress fluid (without thixotropy) can be represented by the Herchel–Bulkley (HB) model, $\tau = \tau _c + k\dot{\gamma }^n$ for $\tau > \tau _c$, where $\tau$ and $\dot{\gamma }$ are the shear stress and the shear rate, respectively, and *k* is a consistency factor. Here, *n* is the rheological exponent and $n=1$ defines a Bingham fluid. However, the Bingham flow in one-dimensional channels can be more complex when considering microstructures in the pores. Nash and Rees (2017) studied one-dimensional flow of Bingham fluid through a bundle of channels with different widths and analytically obtained a wide range of different relations between the Darcy velocity and applied pressure gradient and the thresholds for different distributions of channel widths. Talon et al. (2014) solved analytically the flow of Bingham fluids in one-dimensional rough channel with self-affine aperture variations and obtained non-trivial scalings that depend on aperture openings and the Hurst exponent related to the roughness. These results indicate that a definitive Darcy–Bingham model could not be found for one-dimensional Bingham flow for different channel structures. However, when upscaled to large pore networks, the disorder in pore size distributions and the network topology play dominant role in the macroscopic flow properties. Experimental studies (Al-Fariss and Pinder 1987; Chevalier et al. 2013, 2014) of flow of HB fluid through porous medium have shown the general law between *Q* and $\Delta P$ as $(\Delta P-P_t)\propto Q^n$, where *n* is the same Herchel–Bulkley power law exponent and $P_t$ is an overall threshold pressure drop below which there is no flow through the porous medium. Chevalier et al. (2013) have studied experimentally the flow of different yield-sterss fluids (Carbopol solution and water-in-oil emulsion with $n=0.36$) through a porous media made of glass beads for a wide range of shear rates and proposed an equivalent Darcy’s law by connecting the threshold pressure drop with the system parameters such as bead diameter and fluid yield stress. In a later experiment, they have also reported a breakdown of non-Newtonian characteristics (Chevalier et al. 2014) at the pore level by measuring the local velocity distributions and showing that the yield stress fluid flows in all regions of the porous medium. This was later believed to be due to the high net flow beyond the threshold pressure (Chevalier and Talon 2015). For the case of a Bingham fluid ($n=1$) in a porous media, it was shown numerically (Roux and Herrmann 1987) that there are at-least three different flow regimes exist, $Q\propto (\Delta P-P_t)^\alpha$ with $\alpha$ equal to 1, 2 and 1 when successively increasing the applied pressure drop. This may be understood as follows: the threshold pressure along one continuous flow path throughout the system is the sum of all the thresholds along that path and the minimum sum along all such possible paths corresponds to the global threshold pressure $P_t$ for the system. Just above $P_t$, only one flow path exists, and for constant effective viscosity, $Q\propto (\Delta P-P_t)$ as $n=1$. At higher pressure, more and more flow paths appear, and assuming a linearization of threshold distribution, the increase in the number of paths ($\mathrm{d}N$) due to an increase in the pressure drop $\mathrm{d}\Delta P$ will be $\mathrm{d}N\propto \mathrm{d}\Delta P$ and the corresponding change in the conductance of the network will be $\mathrm{d}\Sigma \propto \mathrm{d}N\propto \mathrm{d}\Delta P$. The increase in the flow rate $\mathrm{d}Q\propto \Sigma \mathrm{d}\Delta P$, and integration over this leads to the quadratic relationship, $Q\propto (\Delta P-P_t)^2$. At higher pressure when all the links are conducting, $\Sigma$ becomes constant and *Q* becomes linear with $\Delta P$. Talon and Bauer (2013) have simulated the flow of Bingham fluids in a stochastic porous media using lattice-Boltzmann simulations and obtained all the three regimes of the flow. With a statistical approach and via lattice-Boltzmann simulations, Chevalier and Talon (2015) measured the exponent as 2.1 for the quadratic regime from the exponents related to the size distributions of nonflowing clusters between flowing channels. Notice that these quadratic and linear dependencies of macro-scale flow rate and excess pressure drop for the intermediate and large pressure regimes of single-phase flow of Bingham fluid are same as that of the two-phase flow of Newtonian fluids (Eq. 2), but the flows at the pore scale are different, for Bingham fluid it is a linear dependence ($n=1$), whereas for two-phase flow of Newtonian fluids it is a square root (Eq. 1).

Experiments of two-phase flow in porous media (Tallakstad et al. 2009a, b; Rassi et al. 2011) have shown this nonlinear quadratic relationship between the flow rate and pressure drop, but the existence of the threshold pressure was not investigated in any previous experiment. Nonlinear flow regimes were also observed for the two-phase flow of non-wetting blobs (droplets) inside a wetting fluid in a high porosity two-dimensional porous media via lattice-Boltzmann simulation (Yiotis et al. 2013) and in experiment (Chevalier et al. 2015). In support of the experimental results observed in the 2D experiment of two-phase flow, a scaling theory was proposed in Tallakstad et al. (2009b) showing $Q\propto \Delta P^2$ without a threshold pressure drop. The scaling theory was based on the assumption that the fluids flow through different channels and there are stuck clusters in between them. The scaling theory is as follows. Let us consider a 2D porous media of width *W* and length *L* where the overall pressure drop as well as the total flow is in the direction of length *L*. There is a number of flow channels $n_l$ in the direction of overall flow. A characteristic length-scale *l* in between the channels is considered and therefore $n_l = W/l$. The flow through each channel is then, $q_c = ka^2\Delta P/(\mu L)$, where *a* is the width and *k* is the permeability of the channel. The total flow through all the channels is then $Q = n_lq_c = ka^2W\Delta P/(\mu lL)$. In order to find the dependence of *l* on $\Delta P$, it is assumed that the stuck clusters in between the channels are held in place by capillary forces $p_c$. If $\lambda$ is the diameter of such a cluster, then the viscous pressure drop around it will be $\lambda \Delta P/L$, which should be $\le p_c$ in order for the cluster to be held in place. Considering $\lambda \Delta P/L = p_c$ for the largest cluster which is not moving and assuming that the separation between the channels (*l*) is the same as the largest non-moving cluster diameter, i.e., $l \approx \lambda =p_c L/\Delta P$, one finds $Q=ka^2W\Delta P^2/(\mu L^2p_c)$ or $Q\propto \Delta P^2$. Though this scaling theory shows the quadratic dependence of *Q* on $\Delta P$, it does not contain any threshold pressure. Moreover, if we extend this theory to three dimensions, the number of channels $n_l$ should be equal to $W^2/l^2$ and following the same arguments we will find $Q\propto \Delta P^3$ for 3D. This is contradictory to the mean-field theory (Sinha and Hansen 2012), according to which the quadratic relationship does not depend of the dimensionality of the network. Secondly, the 3D experiments reported in Rassi et al. (2011) showed that, without considering the threshold pressure, the log–log plot of *Q* and $\Delta P$ shows a variation in the slopes in the range of 0.45–0.3. Those experiments were performed for a short range of capillary numbers and were difficult to draw any definitive conclusions from. It is therefore essential to perform extensive experiments and numerical simulations of two-phase flow in 3D porous medium to find the exact nonlinear dependence of *Q* and $\Delta P$ and to check whether the scaling depends on the with dimensionality of the porous media.

To our knowledge, there is very little experimental work exploring these flows using a three-dimensional pore network. In this article, we present an extensive experimental and numerical study of the steady-state two-phase flow of immiscible Newtonian fluids. In the following, first we will present the experimental study of steady-state two-phase flow of air and deionized water. This work utilizes a three-dimensional porous medium, resulting in a more chaotic flow and increased fluctuations around a mean value. Significant work was done to collect high fidelity data and to accurately obtain the average differential pressures in the steady state. We will then present our numerical work, describing the network model of two-phase flow in three-dimensional reconstructed pore networks. From our experimental and computational results, we will show that there exists a global threshold pressure $P_t$, below which there is no flow through the system. The results are highly significant to understand the steady-state two-phase flow of Newtonian fluids where an effective non-Newtonian behavior emerges from the pore-network topology and the capillary barriers in a disordered system.

## Experimental Setup

**Fig. 1:**
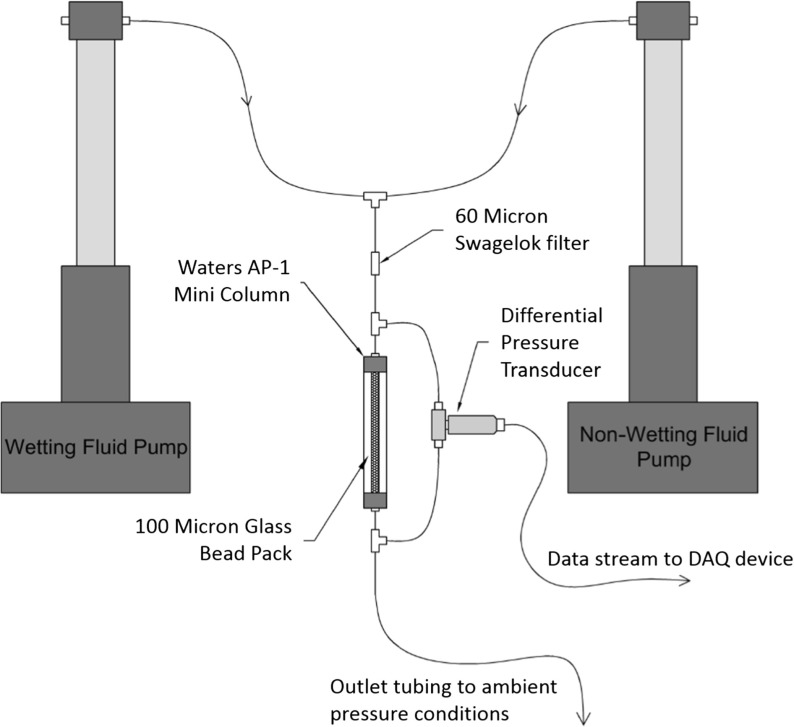
The experimental setup of two-phase flow of deionized water as the wetting fluid and air as the non-wetting fluid through the column of borosilicate glass beads

Experiments were performed on a three-dimensional porous medium confined in a vertical column. The porous medium was comprised of randomly distributed borosilicate glass beads with diameter $d=98\pm 8\,\upmu \text {m}$ and porosity $\phi =0.44$ (Cospheric LLC, Santa Barbara, CA). These microspheres were wet-filled and well packed into a Waters AP-1 Mini Chromatography Column with inner diameter $D=5\,\text {mm}$ and length $L=70\,\text {mm}$. Filters at the inlet and outlet of the column consisted of a bed of glass microfibers and fine plastic mesh. The mesh allowed for uninhibited fluid flow, yet prevented the loss of glass beads. The filters aided in mixing the fluids to further homogenize the two phases before entering the porous media. This was done to emulate the alternating inlet boundary conditions as used in other experiments (Tallakstad et al. 2009b) or simulations (Sinha and Hansen 2012) and to minimize the slug-like flow of alternating fluids. The two immiscible fluids used in this study were deionized water as the wetting fluid, and air as the non-wetting fluid, having respective dynamic viscosities $\mu _\mathrm{w} = 1.002\times 10^{-3}\,\text {Pa}\,\text {s}$ and $\mu _\mathrm{nw} = 1.84\times 10^{-5}\,\text {Pa}\,\text {s}$. This provides the viscosity ratio $M=\mu _\mathrm{nw}/\mu _\mathrm{w}$ equal to $1.83\times 10^{-2}$. The air–water surface tension at room temperature is around 0.073 N/m. The bond number comparing gravitational effects to the surface tension, $E_o=\Delta \rho g d^2/\gamma$ where $\Delta \rho$ is the difference in density of the fluids and *g* the gravitational acceleration, is around 0.001. If we assume the size of an air bubble in the system is of the order of the column diameter $D=5$ mm, the ratio between the buoyancy and the capillary force is $(D/d)E_0\approx 0.05$ making it possible to ignore gravitation in the analysis. We like to point out that the two phases in our experiments, air and liquid, are similar to the experiments by Tallakstad et al. (2009b), where film flow can be neglected. In a similar experiment by Aursjø et al. (2014) with different fluids, a food grade oil and water–glycerol as the two phases, the oil was found to make films in the pores, making a connected system of flowing pathways throughout the whole system, and a large part of the water–glycerol clusters were left behind. The pore-level dynamics is therefore very different in that case, and correspondingly different power law exponents were found for the film-flow system.

Two Teledyne Isco Model 500D syringe pumps were used to control the bulk flow rates of each respective fluid, which were then combined in a junction just above the column inlet. Polyetheretherketone (PEEK) tubing (Sigma-Aldrich Co.) and stainless steel fittings (Swagelok) comprised the flow system, and a sintered stainless steel $60\,\upmu \text {m}$ filter element was used before the inlet to collect any small particulates in the inlet fluids and homogenize the phases. An Omega PX409 differential pressure transducer measured the pressure gradient over the column of glass microspheres with precision and accuracy error below 1%. Pressure data were acquired using National Instruments LabVIEW at a sampling rate of 1000 samples per second, although sample compression was used to average 1000 samples into one data point, yielding one binned data point per second. A schematic of the experimental setup can be seen in Fig. 1.

## Experimental Results and Discussions

Our experimental study is focused on measuring the scaling coefficient $\alpha$ related to the scaling of the flow rate and the excess pressure drop, $(\Delta P-P_t)\propto \text {Ca}^\alpha$, which was shown to be independent of fractional flow, saturation and viscosity ratio (Tallakstad et al. 2009b; Sinha and Hansen 2012). Ca is the capillary number of the system which is a dimensionless parameter describing different regimes of flow, defined as the ratio between the viscous and capillary forces at the pore level and is given by $\text {Ca} = \mu v/\gamma$. Here, $\mu$ is the effective viscosity of the system, $\gamma$ is the surface tension and *v* is the fluid velocity which is equal to the flow rate per cross-sectional pore area. When other parameters are constant, Ca is proportional to the flow rate *Q*. Our experiments use non-wetting fractional flow $F_\mathrm{nw}=0.5$ allowing for the volume of both syringe pumps to be fully utilized. All experimental parameters were kept constant except the flow rates of the fluids, so the overall bulk flow rate was varied initially in order to reach the desired capillary number. This paper focuses on experiments carried out at capillary numbers $10^{-4}$ to $10^{-5.4}$. Before each experiment, the bead pack was initially saturated with water (no air flow) which provided consistency between experiments. The experiments were conducted by simultaneously starting the flow of both water and air at the same flow rate, resulting in the desired non-wetting fractional flow of $F_\mathrm{w}=0.5$. The total flow rate *Q* for any given capillary number Ca is obtained from the fluid velocity *v* given by $v=\gamma \text {Ca}/\mu$, and then multiplying it with the effective cross section *A*, $Q=Av$. Here, $\mu$ is the effective viscosity at room temperature. The effective cross-sectional area *A* is obtained from the total cross-sectional area of the bead pack multiplied with its porosity ($\phi =0.44$). The volumetric flow rate for each fluid (air and water) is then half of the calculated total flow rate *Q*. Due to the compressibility of the air, the phases likely competed at the junction, causing some degree alternating injections of water and air. Again, the filter was utilized as a means to reduce this phenomenon and create a more homogenized flow into the bead pack. The non-wetting saturation of the bead pack increased as air became randomly distributed throughout the porous medium. The confining column was borosilicate glass which provided for basic visual observations of the bead pack saturation.

**Fig. 2:**
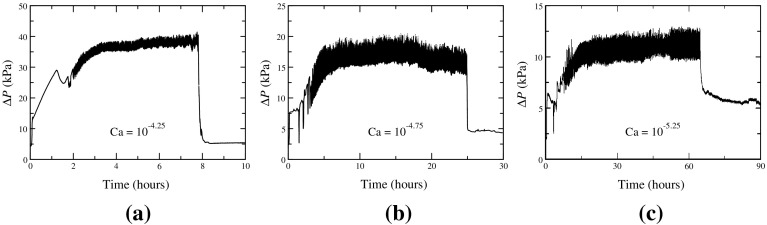
Three sample pressure plots of the time evolution of the experiment are shown to demonstrate the chaotic and transient nature of the two-phase flow. Plots from the high-Ca regime (**a**), transition point (**b**), and low-Ca regime (**c**) illustrate the characteristic pressure behavior observed

In Fig. 2, pressure profiles are plotted over the duration of experiments at respective Ca. The pressure gradient during the transient regime followed a chaotic, increasing trajectory. Reaching the steady-state regime, the pressure fluctuated around an average differential pressure $\Delta P$. Capillary forces between the competing phases manifest in large fluctuations of $\Delta P$ even at lower Ca values. The $\Delta P$ fluctuations maintain the same overall magnitude and therefore more pronounced in the low-Ca regime when the absolute value of $\Delta P$ is lower. Upon stopping the pumps, we have $Q=0$; however, the pressure gradient over the column fell to a nonzero value which we identify as the threshold pressure $P_t$. We were able to obtain experimental values for $P_t$ that slightly differed between experiments and took the average of these values as a global threshold pressure for the column, $P_t=5.39$ kPa.

**Fig. 3:**
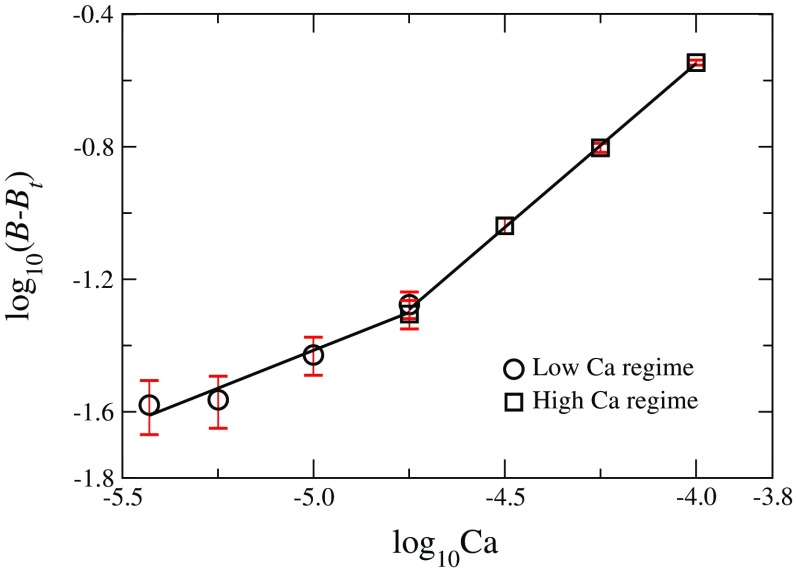
Plot of dimensionless excess pressure drop ($B-B_t$) as a function of the capillary number (Ca) for the nonlinear and linear flow regimes obtained from the experiment. Each *data point* represents the averaged steady-state pressure gradient for the respective Ca. The *error bars* are obtained from the fluctuations in the pressure drops in the steady state. The two scaling exponents for the low and the high-Ca regimes obtained from the slopes are $0.46\pm 0.05$ and $0.99\pm 0.02$ respectively

The excess pressure drops $(\Delta P-P_t)$ are normalized to the dimensionless pressures (Chevalier et al. 2015) defined as $B=D^2|\nabla P|/\gamma$, where $\nabla P =\Delta P/L$. In Fig. 3, they are plotted as a function of Ca where two flow regimes corresponding to two different slopes can be observed. For capillary numbers $10^{-4.75}$ and higher, the excess pressure drops scale nearly linearly with Ca with $\alpha =0.99$ whereas at low capillary numbers, the relationship is nonlinear with the exponent $\alpha =0.46$. These values support the power scaling theory from Sinha et al. and demonstrate that incorporating $P_t$ is crucial to obtain a consistent scaling factor of $\alpha = 0.46$. There is a distinct change from the Newtonian to non-Newtonian flow regime at $\text {Ca}=10^{-4.75}$; however, this transition point is far from the values found by similar experimental and numerical studies performed in two-dimensions (Tallakstad et al. 2009a; Sinha and Hansen 2012), as it depends on the porous media and other flow parameters.

The small number of data points in our experiment is due to the restriction imposed by an aging effect observed in our porous medium which made the experiments challenging, as all data must be collected before the aging of the column. In the initial trials of our experiments, we found that the magnitude of $\Delta P$ required to maintain a specific two-phase flow rate goes higher if a time span of several weeks were elapsed between measurements, yet all the data collected within a short time frame would show a scaling of 0.4–0.5 in the low-Ca regime, or 0.95–1.05 in the high-Ca regime. Some of this was explained initially by biofouling of the inline filter and mesh, which was avoided by adding a small amount of biocide to the flowing fluid. In two-phase flow experiments with a Hele-Shaw cell, Aursjø et al. observed this aging effect phenomena and theorized that it was due to wetting effects (Aursjø et al. 2014). We hypothesize that similar wetting effects within the column of glass microspheres may have contributed to the change of the non-wetting saturation $S_\mathrm{nw}$, resulting in a different $\Delta P$ for a given two-phase flow rate. This theory is corroborated by the simulations which show $\Delta P$ is a function of $S_\mathrm{nw}$ (see Fig. 7). This aging imposed the restriction on the experimental time span, and the final data set presented here was performed over the smallest time span possible, in order to avoid all aging effects. As shown in Fig. 2c, it can take as long as 4 days to obtain one data point. The full set of 8 experimental data points presented here were arranged to take place in a 2 week period, and the data point at $\log _{10}\text {Ca} = 4.75$ was repeated at the end of the experimental run to confirm that no aging had occurred.

**Table 1:** Physical dimensions of the porous media samples A, B and C and the number of links and nodes of the corresponding reconstructed networks

Network	Physical dimension ($\text {mm}^3$)	Number of nodes	Number of links
A	$1.8\times 1.8\times 1.8$	1163	2274
B	$4.5\times 1.5\times 1.5$	767	1750
C	$1.0194\times 1.0194\times 1.0194$	604	1054

## Network Model for Two-Phase Flow in Reconstructed Pore Network

Network models of multiphase flow are useful to understand the macroscopic properties of large pore networks relating the underlying pore-scale physics of a porous material (Niasar and Hassanizadeh 2012). A few decades ago, medical micro-CT (microcomputed X-ray tomography) scanners were adopted to scan geological samples (Hurst 1984) and since then there has been a revolution in the new scanning techniques to characterize the microstructures of porous media (Dunsmoir et al. 1991; Blunt et al. 2013). To use these pore structures obtained from the 3D images of the pore space as the input to the network models, simplified networks consisting of pores and throats are reconstructed using different approaches. One approach is to use a statistical model (Okabe and Blunt 2005) where different statistical properties like the porosity distribution, correlation function and linear path function are estimated from the images of 2D thin sections of the sample. Random 3D networks are then generated with the same statistical properties obtained from the images. This method is often questioned and observed to match poorly with the original sample due to the loss of long range geometric connectivity (Manwart et al. 2000; Jiao et al. 2009; Øren and Bakke 2002). Another method of reconstruction is by *process-based* models, where 2D thin section images are analyzed to measure the grain size distribution and other petrophysical properties and then the packing of the grains are simulated following different geological processes, such as sedimentation, compaction, rearrangement and diagenesis (Øren and Bakke 2002, 2003). These models show good results for samples where sedimentary processes are involved, such as sandstones. However, in the case of systems having complex sedimentary and diagenetic history or systems with heterogeneity—such as carbonates—network reconstruction using process-based models are difficult. In such cases, to extract the pore networks from any generic 3D image of an arbitrary porous medium, different voxel-based models such as medial axis-based methods and maximum ball methods are used (Dong 2007; Dong and Blunt 2009). In the present study, we use three different networks reconstructed from (A) Berea sandstone, (B) sandpack and (C) a sandstone [“sandstone 9” in Dong (2007)]. Networks A and B were reconstructed using the process-based models (Øren and Bakke 2002, 2003), whereas C was reconstructed using a voxel-based maximum ball algorithm (Dong 2007; Dong and Blunt 2009). The physical dimension of the samples A, B and C and the number of links and nodes of the corresponding reconstructed networks are listed in Table 1.

Sample A and B have been used previously, e.g., in Ramstad et al. (2009) and Tørå et al. (2012), respectively. Samples C is described in Dong (2007) and may be found at www.imperial.ac.uk/earth-science/research/research-groups/perm/research/pore-scale-modelling/micro-ct-images-and-networks/sandstone-s9/.

**Fig. 4:**
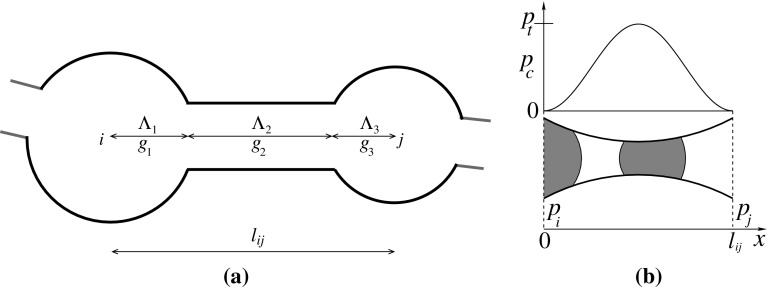
The schematic of one link between the two nodes *i* and *j* is shown in (**a**). The pore space of each link is divided into three pore parts, two pore bodies at two ends and one pore throat in between. The total length ($l_{ij}$) of the link is equal to $\varLambda _1+\varLambda _2+\varLambda _3$, the sum of each pore part. The presence of multiple interfaces between the wetting (*white*) and non-wetting (*gray*) fluids in a link is shown in the bottom of (**b**), and the variation of capillary pressure $p_c(x)$ as a function of the interface position (*x*) for each interface is shown in the top of (**b**). $p_c=0$ at the two ends of the link and is maximum, equal to the threshold pressure $p_t$, at the middle of the tube. $p_t=4\gamma \cos \theta /r_t$ is the minimum pressure required for the non-wetting fluid to invade the pore

The network extracted from a pore sample consists of links that are connected at nodes. The number of links connected to a node is the degree or the coordination number of that node. Each link is associated with a set of parameters which characterize the pore space of the original sample. A link has three pore parts, two pore bodies at the end and one pore throat in between as shown in Fig. 4. The cross section of the pores is triangular in shape and characterized by a shape factor *G*, defined as the ratio between the effective cross-sectional area of the pore (*a*) and the square of its circumference. The value of *G* can vary in the range $(0,\sqrt{3} /36]$ for triangular cross section where the largest value ($\approx$0.048) corresponds to an equilateral triangle. The effective cross-sectional area can then be calculated from the relation $a=r^2/(4G)$, where *r* is the radius of the inscribed circle in the pore part (Mason and Morrow 1991). The network transports two immiscible fluids, one is more wetting than the other with respect to the pore wall. We pointed out in the experimental section that the wetting properties of the fluids are such that we consider no film flow in the system and the flow in the pores are piston-like. The instantaneous local flow rate $q_{ij}$ inside a link between two nodes *i* and *j* follows the Washburn equation of capillary flow (Washburn 1921; Aker et al. 1998),

3 $$\begin{aligned} \displaystyle q_{ij}= \frac{g_{ij}}{l_{ij}}\left[ p_j-p_i-\sum p_c(x)\right] , \end{aligned}$$

where $p_i$ and $p_j$ are the local pressure drops at *i*th and *j*th nodes. The mobility $g_{ij}$ of the link between the two nodes is calculated from the harmonic average of the individual conductances from each of three pore parts of that link, given by,

4 $$\begin{aligned} \displaystyle \frac{l_{ij}}{g_{ij}}=\frac{\varLambda _1}{g_1}+ \frac{\varLambda _2}{g_2}+\frac{\varLambda _3}{g_3}, \end{aligned}$$

where $\varLambda _{1,2,3}$ and $g_{1,2,3}$ are the lengths and conductances of each pore part, respectively, as shown in Fig. 4. For a triangular cross section, each individual term of $g_{1,2,3}$ for each pore part of a link is given by Langglois (1964) and Jia et al. (2008),

5 $$\begin{aligned} \displaystyle g = \frac{3r^2a}{20\mu _p}, \end{aligned}$$

where *a* and *r* are, respectively, the effective area and the radius of the inscribed circle in the pore part. $\mu _p$ is the time-dependent saturation-weighted viscosity for the link given by $\mu _p=\mu _\mathrm{nw}s+\mu _\mathrm{w}(1-s)$, where *s* is the instantaneous non-wetting saturation inside the link.

The capillary pressure $p_c(x)$ in Eq. 3 appears due to the surface tension at the interfaces where $x\in [0,l_{ij}]$ is the position of the interface. It acts as a barrier for the non-wetting fluid to penetrate through the pores filled with the wetting fluid and will be maximum at the narrowest part of the pore, i.e., the pore throat. The pores are in between grains and the links are therefore approximated as hourglass-shaped in the longitudinal direction in terms of the capillary pressure as shown in Fig.4(b). The functional dependence of the capillary pressure on the interface position inside such a pore is modeled by a modified form of Young-Laplace equation (Dullien 1992; Aker et al. 1998),

6 $$\begin{aligned} \displaystyle |p_c(x)| = \frac{2\gamma \cos \theta }{r_t}\left[ 1-\cos \left( \frac{2\pi x}{l}\right) \right] , \end{aligned}$$

where $r_t$ denotes the throat radius which is the narrowest part of the pore. $\gamma$ is the surface tension, and $\theta$ is the contact angle between the interface and the pore wall. In our simulations, we set $\gamma \cos \theta = 0.03$ N/m. The chosen form of $p_c(x)$ therefore provides the necessary *x*-dependence so that $p_c(0)=p_c(l)=0$ and $\displaystyle \max _{x\in [0,l_{ij}]}|p_c(x)| = |p_c(l_{ij}/2)|$. The summation over $p_c$ in Eq. 3 runs over all the interfaces inside one link.

A constant volumetric flow rate *Q* is generated through the application of a pressure drop $\Delta P$ across the system. Local pressures ($p_i$) at each node are then determined by solving the set of linear equations balancing the flow at each node using the Kirchhoff equations, ensuring that the net flux in any node is zero. This is done by solving the corresponding matrix inversion problem using the conjugate gradient algorithm (Batrouni and Hansen 1988). When the local node-pressures are known, the local flow rates $q_{ij}$ through each link is calculated using Eq. 3. This determines the velocity of each interface inside any link. We choose an adaptive time step $\Delta t$ in such a way that the displacement of any meniscus does not exceed one-tenth of the length of the corresponding link within that time. In the regime of the capillary numbers here, the Reynolds numbers at the pores are much smaller than 1 and we therefore assume piston-like creep flow in the links. All the interfaces are moved accordingly which changes the pressure distribution in the network. The pressures at the nodes are then determined again using conjugate gradient algorithm, and the whole process is repeated. When an interface reach at the end of a link, wetting and non-wetting bubbles are snapped-off and new interfaces are created in the neighboring links. The rules related to the interface dynamics used in this 3D network model are similar to the 2D network model in Aker et al. (1998) and Knudsen et al. (2002). The in- and out-fluxes of the fluids from one node to the neighboring links at any time step are determined from the relative flow rates of the links connected to that node. As this can increase the number of interfaces in any link infinitely, we put a limit in the maximum number of interfaces inside any link. When this limit is exceeded, we merge the two nearest interfaces keeping the volume of each fluid conserved. In the simulations reported in this article, we have considered a maximum of 4 interfaces in any link.

In order to reach steady state, we need to impose the periodic boundary condition so that the fluid configurations that leave the network from one side can enter from the opposite side and the flow can continue for infinite time. However, the network here is irregular and therefore the two opposite edge surfaces do not match each other which is necessary to apply the periodic boundary condition. We solve this problem by making a mirror copy of the network in the direction of the overall flow and connected the copy with the original network. The edges then match each other, and periodic boundary conditions are implemented in the direction of overall pressure gradient. Notice that this makes the system closed, and the fluid saturations do not change with time. The saturation is therefore a control parameter here, and we measure the fractional flow, whereas in our experiments we control the fractional flow.

## Simulation Results

**Fig. 5:**
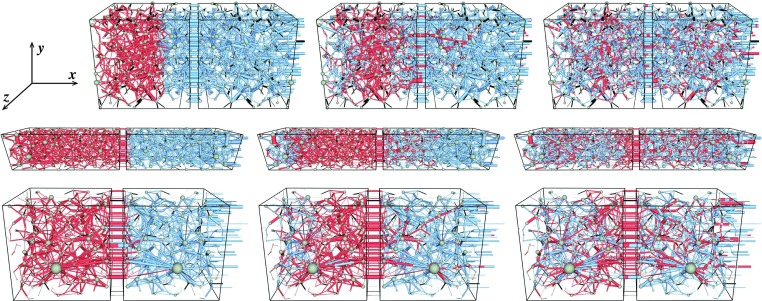
The time evolution of two-phase flow through the reconstructed networks where the wetting and the non-wetting fluids are colored by *blue* and *red*, respectively. The few links in *black* are the dead ends which are connected only at one node and therefore removed from the network. The three images from *left* to *right* in each row, respectively, correspond to the initial condition of the system and after 0.1 and 0.3 pore volumes of fluids have passed. The three rows from *top* to *bottom* correspond to the samples A (berea), B (sandpack) and C (sandstone), respectively. The overall flow is in the positive *x* direction. The periodic boundary condition is implemented in the same direction, by making a mirror image of the original reconstructed network and then connected together with the original. These two parts are shown by the two cuboids in the figures. Here, the system is initialized by filling the links with non-wetting fluid from $x=0$ until the required saturation is obtained and then filling the rest with the wetting fluid. In these figures, the non-wetting saturations are 0.3 for sample A and 0.5 for B and C

Simulations are performed with constant flow rate *Q*, which sets the capillary number Ca, the ratio of the viscous to the capillary forces at the pore level, given by $\text {Ca}=Q\mu /(\gamma A)$. Here, *A* is the cross-sectional area of the pore space, $\mu$ is the saturation-weighted viscosity and $\gamma$ is the surface tension between the two fluids. We have considered two viscosity ratios, $M=1$ where $\mu _\mathrm{nw} = \mu _\mathrm{w} = 0.1\,\text {Pa}\,\text {s}$ and $M=0.1$ where $\mu _\mathrm{nw} = 10^{-3}\,\text {Pa}\,\text {s}$ and $\mu _\mathrm{w}=10^{-2}\,\text {Pa}\,\text {s}$. For a reservoir, the oil/water viscosity ratio can vary in a wide range depending on the type of the oil and the temperature (Barillas et al. 2008). Initial transients during the simulation at $\text {Ca}=0.01$ and $M=1$ are shown in Fig. 5 where the wetting and non-wetting fluids are colored by blue and red, respectively. There is a few links marked by black. These are the links which are connected only with one node and will act as a dead end. The three rows from top to bottom correspond to the three networks A, B and C, respectively, and the three snapshots from left to right for each network are taken at three different time steps—at the beginning of the simulation and after 0.1 and 0.3 pore volumes of fluid have passed. The overall flow is in the positive *x* direction. There are two cuboids in each network, the one inside the left cuboid is the original reconstructed network and in the right one is the mirror copy of that, which is done in order to implement the periodic boundary condition as discussed earlier. We prepared the initial system by filling it sequentially by two fluids with necessary saturation so that the network is segregated into one part of non-wetting (red) fluid and one part of wetting (blue) fluid as shown in Fig. 5. Here $S_\mathrm{nw}=0.3$ for A and 0.5 for B and C. When the simulation starts, the non-wetting fluid starts invading the wetting fluid and depending on the capillary number and viscosity ratio it will either start viscous fingering for high Ca or the capillary fingering when the capillary forces dominate. On the other hand, the wetting fluid also enters the system from left due to the periodic boundary and pushes the non-wetting fluid. This displacement of wetting fluid into the non-wetting is favorable, and the displacement should be piston-like or stable displacement. Though the network is small here, one can still observe some signatures of these two different types of fluid displacements in the initial transients—in the right part, there are capillary fingers of the red fluid into the blue, whereas in the left the blue fluid displaces the red more uniformly with compact propagating front. Detailed time evolution of the fluid displacements during the initial transients are presented in the animations in the supplementary material. The non-wetting saturation, capillary number and instantaneous pore volumes of fluids passed are indicated in the respective animations.

**Fig. 6:**
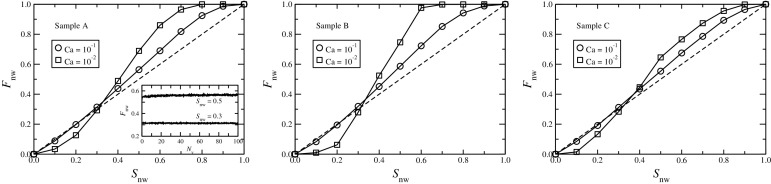
Plots of non-wetting fractional flow ($F_\mathrm{nw}$) in the steady state as a function of non-wetting saturation ($S_\mathrm{nw}$) for the three different networks. The *dashed diagonal straight lines* correspond to $F_\mathrm{nw}=S_\mathrm{nw}$, a system of miscible fluids will follow that *line*. The results for two different capillary numbers, $\text {Ca} = 10^{-1}$ and $10^{-2}$, are shown. Notice that the curves approach the diagonal *straight line* for the higher value of Ca. In the *inset* for sample A, we plot the fractional flow as a function of pore volumes ($N_\mathrm{v}$) of fluids passed, where $F_\mathrm{nw}$ fluctuates around an average value in the steady state

The two fronts catch up with each other with time, and the system eventually contains a mixture of two fluids in the steady state. In the steady state, both drainage and imbibition take place at the pore level and fluid clusters are created, merged and broken up. We identify the steady state when the average of any measurable macroscopic quantity stops drifting with time and starts fluctuating around a constant average value. Instead of the sequential initial condition shown in Fig. 5, where the two fluids are segregated at two parts of the system, we may start the simulation from a random initial condition where the two fluids are distributed randomly. In that case, we found that the simulations reach to the steady state much faster. Therefore, in order to save the computational time we adopted the random initial condition in all the following results. When the system evolves into the steady state, we measure the macroscopic properties with time and take averages of the measurements.

**Fig. 7:**
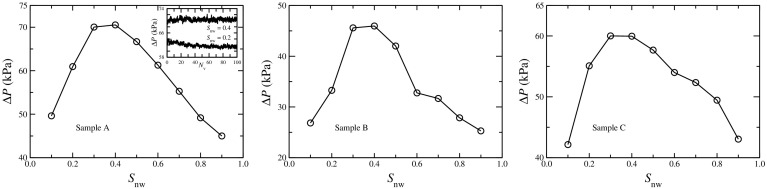
Total pressure drop ($\Delta P$) in the steady state for the three networks as a function of $S_\mathrm{nw}$. The capillary number $\text {Ca} = 10^{-2}$. $\Delta P$ reaches a maximum at an intermediate saturation, which is due to the increasing number of interfaces causing higher capillary barriers. In the *inset* of sample A, $\Delta P$ is plotted as a function of the pore volumes of fluids passed, which shows the evolution of steady state

First, we present some fundamental properties of the system by measuring the non-wetting fractional flow ($F_\mathrm{nw}$) and the pressure drop across the system ($\Delta P$) as a function of the saturation. The non-wetting fractional flow is defined as the proportion of non-wetting fluid flowing across the system given by $F_\mathrm{nw} = Q_\mathrm{nw}/Q$ where $Q_\mathrm{nw}$ is the volumetric flow rate of non-wetting fluid through any network cross section perpendicular to the overall flow. For a set of miscible fluids with no capillary forces, $F_\mathrm{nw}$ will be exactly equal to $S_\mathrm{nw}$ in the steady state for any saturation, which is not the case here. The measurements of $F_\mathrm{nw}$ in the steady state are illustrated in Fig. 6. In the inset of the first plot, we show the variation of $F_\mathrm{nw}$ as a function of the pore volumes of fluids passed through the system which ensures the steady-state flow situation. The time averages of $F_\mathrm{nw}$ in the steady state for the whole range of non-wetting saturation are then plotted for the three different networks at two capillary numbers $\text {Ca}=10^{-1}$ and $10^{-2}$. The plots show the well known S-shape and do not follow the diagonal dashed line corresponding to $F_\mathrm{nw}=S_\mathrm{nw}$. This is due to the presence of capillary forces at the interfaces for which the two immiscible fluids do not flow equally. The one with the higher saturation dominates the flow, $F_\mathrm{nw}$ is less than $S_\mathrm{nw}$ for low values of $S_\mathrm{nw}$ and higher than $S_\mathrm{nw}$ for higher values of $S_\mathrm{nw}$. The curves therefore cross the $F_\mathrm{nw} = S_\mathrm{nw}$ line at some point, which is not at $S_\mathrm{nw}=0.5$. This shows the asymmetry between the two fluids, as one fluid is more wetting than the other with respect to the pore walls. Moreover, a lower capillary number corresponds to stronger capillary forces relative to the viscous forces and hence the deviation of the curves from the diagonal straight line is higher for $\text {Ca}=10^{-2}$ than for $10^{-1}$ for each network.

Variation of the global pressure drop $\Delta P$ as a function of the saturation $S_\mathrm{nw}$ for constant overall flow rate (*Q*) is shown in Fig. 7 for the three networks. Here, the capillary number $\text {Ca}=10^{-2}$ for these plots. The initiation of the steady state is illustrated in the inset of sample A, where $\Delta P$ fluctuates around an average value in the steady state. The average of $\Delta P$ in the steady state first increases with the saturation, reaches a maximum and then decreases again. When $S_\mathrm{nw}$ is increased from zero, the single-phase flow regime, more interfaces start appearing into the system. This increases the overall capillary barrier and to keep the same flow rate (*Q*) the pressure drop across the system needs to be increased. Therefore, $\Delta P$ is maximum at some intermediate saturation and then starts decreasing again as $S_\mathrm{nw}$ approaches to 1, as the system again approaches to the single-phase flow regime. Interestingly, the maximum of $\Delta P$ is not at $S_\mathrm{nw}=0.5$, but close to the saturation where $F_\mathrm{nw}$ crosses the diagonal $F_\mathrm{nw}=S_\mathrm{nw}$ line as observed in Fig. 6. This was predicted in Hansen et al. (2016).

**Fig. 8:**
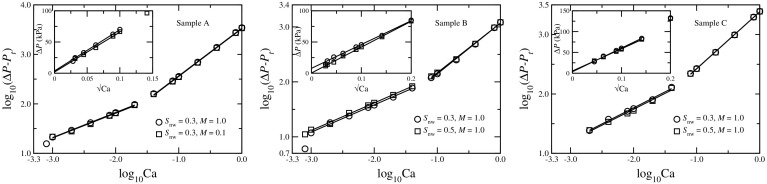
Plot of overall pressure drop as a function of the capillary number in the steady state for different networks. For network A, results are shown for $S_\mathrm{nw}=0.3$ with two different viscosity ratios $M=1.0$ and 0.1. For B and C, two different saturations $S_\mathrm{nw}=0.3$ and 0.5 with $M=1.0$ are considered. Measurements of the threshold pressures for each simulation are shown in the *insets* of *each figure*, where $P_t$ is obtained from the *y*-axis intercepts of $\Delta P$ versus $\sqrt{\text {Ca}}$ plots. Using the values of $P_t$, the scaling exponents are then obtained from the slopes of log–log plots of ($\Delta P-P_t$) versus Ca

We now present the simulation results related to the scaling of $\Delta P$ with *Q* in the steady state. The values of Ca range from $\approx 10^{-3}$ to 1 in our simulations. We observed that the crossover from the nonlinear to the linear scaling falls in this range, and we can see both the scaling regimes. Results are illustrated in Fig. 8 where we plot $\log (\Delta P-P_t)$ as a function of $\log \text {Ca}$. First, we need to find the threshold pressure $P_t$ for each case. In our experiments, $P_t$ was found by measuring the differential pressure at $Q=0$. In the simulations for the Bingham fluid (Roux and Herrmann 1987), $P_t$ was determined by decreasing the external current (or flow rate) from a large value and identifying the flow paths with a search algorithm. This procedure is not feasible for dynamic two-phase flow network models, as the interfaces move with time and consequently the flow paths change. In the case of the two-phase flow simulations in a 2D regular network (Sinha and Hansen 2012), $P_t$ was measured by minimizing the linear least-square fit errors of $\log (\Delta P-P_t)$ versus $\log \text {Ca}$ data. There, the numerical results were averaged over different samples and time, whereas in the case of the reconstructed 3D network we only have one network per sample and do not have opportunity to do sample averaging. This leads to higher statistical fluctuations in the error measurement and therefore finding $P_t$ based on the minimum error was not possible. Therefore, as $P_t$ is the value of $\Delta P$ as $Q\rightarrow 0$, we calculated $P_t$ directly from the numerical data of $\Delta P$ versus *Q*. For the low-Ca regime, we expect $Q\propto (\Delta P-P_t)^2$ and therefore we plot $\Delta P$ as a function of $\sqrt{\text {Ca}}$ which leads to a straight line for each sample. The plots are shown in the insets in Fig. 8. Values of the intercepts of the straight lines in the *y*-axis correspond the threshold pressure $P_t$. Using these values of $P_t$, we plot $\log (\Delta P-P_t)$ as a function of $\log \text {Ca}$ in Fig. 8 for the three samples for different values of $S_\mathrm{nw}$ and different viscosity ratios. From the slopes, we see two distinct regimes of flow with two different slopes and there is a sharp crossover in between the two regimes. The results are summarized in Table 2. For the low-Ca regime, all the slopes are close to 0.5 which lead to $Q\propto (\Delta P-P_t)^2$ as shown in Eq. 2. For the high flow rates, all the slopes are close to 1 and the flow is Newtonian.

**Table 2:** Values the threshold pressures $P_t$ and the corresponding scaling exponents $\alpha$ for different simulations

Sample	*M*	$S_\mathrm{nw}$	$P_t$ (kPa)	$\alpha$ (Low Ca)	$\alpha$ (High Ca)
A	1.0	0.3	3.543	$0.51\pm 0.01$	$0.96\pm 0.01$
	0.1	0.3	1.745	$0.50\pm 0.02$	$0.97\pm 0.01$
B	1.0	0.3	7.795	$0.50\pm 0.01$	$0.92\pm 0.02$
	1.0	0.5	0.343	$0.50\pm 0.01$	$0.90\pm 0.02$
C	1.0	0.3	3.354	$0.54\pm 0.02$	$0.96\pm 0.01$
	1.0	0.5	5.183	$0.54\pm 0.03$	$0.96\pm 0.01$

The threshold pressures are obtained from the intercepts in the *y*-axis of $\Delta P$ versus $\sqrt{\text {Ca}}$ plots as shown in the insets of Fig. 8. Using the values of $P_t$, the scaling exponents are then obtained from the slopes of $\log (\Delta P-P_t)$ versus $\log \text {Ca}$ plots

An interesting aspect we observe concerns the transition point from the nonlinear low-Ca regime to the linear high-Ca regime. This transition point seems to vary between studies. In the experiments, we find it around $\text {Ca}\approx 10^{-4.75}$, while our simulations show it around $\text {Ca}\approx 10^{-1.5}$. Even in the simulations, the transition point seems to vary slightly among different porous media samples A, B, and C. Furthermore, Tallakstad et al. did not observe the transition for $\text {Ca}<10^{-1}$ and therefore it should be at a more higher value (Tallakstad et al. 2009a). It seems that the transition point is strongly determined by geometric and physical characteristics of the porous medium itself. At this point, we cannot answer how this transition point depends on the network characteristics, saturation or viscosity ratio and will be an interesting aspect to explore in the future.

## Conclusions

In this article, we presented experimental and numerical studies to investigate the relationship between the pressure drop and the volumetric flow rate in the steady-state two-phase flow of immiscible fluids in three-dimensional porous media. Our two-phase flow experiments utilize a three-dimensional porous medium made of glass beads with air and deionized water flowing through it. For the numerical simulations, we constructed network model transporting two immiscible fluids in three-dimensional reconstructed pore networks. Our experimental and numerical results show that the capillary pressures at the interfaces in between the immiscible fluids introduce pressure barriers at the pores and effectively create a yield threshold in the system, making the fluids to behave like a Bingham viscoplastic fluid in a network. Two flow regimes with quadratic and linear dependencies of flow rate over the excess pressure drop are explored, which are similar to the intermediate and high pressure regimes of single-phase Bingham flow in porous media. These results are in the agreement with the two-dimensional numerical results and the mean-field theory of two-phase flow (Sinha and Hansen 2012), and show that the quadratic scaling does not depend on the dimensionality of the pore network. However, as there are at least three flow regimes exist in the single-phase flow of Bingham fluid in porous media (Roux and Herrmann 1987; Talon and Bauer 2013), one more flow regime may also exist for the two-phase flow of Newtonian fluids in porous media at a more lower value of Ca, which corresponds to the flow in single flow channel. Three flow regimes were also observed for the two-phase flow of non-wetting blobs (droplets) inside wetting fluid in a two-dimensional porous media with high porosity (Yiotis et al. 2013; Chevalier et al. 2015). So far, we could not explore this very low pressure drop regime for the pore-network models due to extreme slow convergence to the steady state, and the rheology in that regime is an open problem to investigate.

We have considered $S_\mathrm{nw}=0.3$ and 0.5 in the simulations and $F_\mathrm{nw}=0.5$ in the experiments which are in the intermediate range of saturation or fractional flow. In this regime, both the fluids contribute to the flow and a large number of interfaces exist in the system introducing capillary barriers at each pore. If one moves toward $S_\mathrm{nw}\rightarrow 0$ or 1, the system will approach to the single-phase flow regime and eventually the interfaces as well as the capillary barriers will disappear. This should drive the system completely into the linear Newtonian flow regime. The quadratic flow regime we have seen here should therefore disappear as $S_\mathrm{nw}\rightarrow 0$ or 1, and the linear flow regime will cover the whole range of Ca. It will be interesting to study this transition as a function of the saturation or the fractional flow by exploring the whole parameter space. However, this needs an enormous range of experiments and simulations as approaching steady state needs many pore volumes of the fluid to be reached. Recently, a Monte Carlo (MC) algorithm has been proposed for the network models of two-phase flow in 2D networks (Savani et al. 2017) and we look forward toward further development of the MC algorithm for the 3D networks in order to have an efficient way to study the effective scaling of the pressure drop and the flow rate of steady-state two-phase flow for the whole parameter space. Finally, we like to mention that, utilizing the property that the thresholds of the yield stress fluids are directly related to the pore sizes, there are inverse techniques to calculate the pore size distributions from the injection experiment data of yield stress fluids (Castro et al. 2014). Because of the existence of the similar thresholds in the two-phase flow of Newtonian fluids, similar kind of inverse techniques may be developed to find the pore size distributions from the experimental results of two-phase flow of Newtonian fluids in porous media.

## Electronic supplementary material

Below is the link to the electronic supplementary material.

Supplementary material 1 (mp4 89813 KB)

Supplementary material 2 (mp4 137308 KB)

Supplementary material 3 (mp4 98119 KB)

## Footnotes

1 Steady state signifies that the macroscopic parameters describing the flow remain constant. It does *not* imply that the interfaces in the pores do not move, split or merge.
